# Immunologic Effects of Stereotactic Body Radiotherapy in Dogs with Spontaneous Tumors and the Impact of Intratumoral OX40/TLR Agonist Immunotherapy

**DOI:** 10.3390/ijms23020826

**Published:** 2022-01-13

**Authors:** Mary-Keara Boss, Remy Watts, Lauren G. Harrison, Sophie Hopkins, Lyndah Chow, Erin Trageser, Carina Easton, Susan M. LaRue, Daniel Regan, Mark W. Dewhirst, Steven Dow

**Affiliations:** 1 Department of Environmental Health and Radiological Sciences, Colorado State University, Fort Collins, CO 80523, USA; lauren.harrison@colostate.edu (L.G.H.); erin.trageser@colostate.edu (E.T.); susan.larue@colostate.edu (S.M.L.); 2 Department of Companion Animals, Atlantic Veterinary College, Charlottetown, PE C1A 4P3, Canada; rwatts5391@upei.ca; 3 Department of Clinical Sciences, Colorado State University, Fort Collins, CO 80523, USA; leone.hopkins@colostate.edu (S.H.); Lyndah.chow@colostate.edu (L.C.); steven.dow@colostate.edu (S.D.); 4 Department of Microbiology, Immunology, and Pathology, Colorado State University, Fort Collins, CO 80523, USA; carina.easton@colostate.edu (C.E.); daniel.regan@colostate.edu (D.R.); 5 Department of Radiation Oncology, Duke University Medical Center, Durham, NC 27710, USA; mark.dewhirst@duke.edu

**Keywords:** dog, cancer, T cells, cytokines, Toll-like receptor, macrophage

## Abstract

Stereotactic body radiotherapy (SBRT) is known to induce important immunologic changes within the tumor microenvironment (TME). However, little is known regarding the early immune responses within the TME in the first few weeks following SBRT. Therefore, we used the canine spontaneous tumor model to investigate TME responses to SBRT, and how local injection of immune modulatory antibodies to OX40 and TLR 3/9 agonists might modify those responses. Pet dogs with spontaneous cancers (melanoma, carcinoma, sarcoma, n = 6 per group) were randomized to treatment with either SBRT or SBRT combined with local immunotherapy. Serial tumor biopsies and serum samples were analyzed for immunologic responses. SBRT alone resulted at two weeks after treatment in increased tumor densities of CD3+ T cells, FoxP3+ Tregs, and CD204+ macrophages, and increased expression of genes associated with immunosuppression. The addition of OX40/TLR3/9 immunotherapy to SBRT resulted in local depletion of Tregs and tumor macrophages and reduced Treg-associated gene expression (FoxP3), suppressed macrophage-associated gene expression (IL-8), and suppressed exhausted T cell-associated gene expression (CTLA4). Increased concentrations of IL-7, IL-15, and IL-18 were observed in serum of animals treated with SBRT and immunotherapy, compared to animals treated with SBRT. A paradoxical decrease in the density of effector CD3+ T cells was observed in tumor tissues that received combined SBRT and immunotherapy as compared to animals treated with SBRT only. In summary, these results obtained in a spontaneous large animal cancer model indicate that addition of OX40/TLR immunotherapy to SBRT modifies important immunological effects both locally and systemically.

## 1. Introduction

Radiotherapy (RT) can serve as an immune stimulus, as various activated immune cells are recruited to the tumor microenvironment following radiation exposure [1,2]. Stereotactic body radiotherapy (SBRT) provides high dose, high precision radiotherapy in a few fractions; SBRT differs from conventionally fractionated RT protocols, which span several weeks. Anti-tumor immune responses may be enhanced when radiation therapy is combined with immunotherapy [3,4,5,6,7,8]. SBRT dosing can be highly immunogenic, eliciting increased antigen presentation and activation of CD4 and CD8 T cells, particularly when combined with immunotherapy [7,8]. Emerging preclinical and clinical data suggest that the combination of SBRT with immunotherapy may be particularly effective in converting immunologically “cold” tumors into immunologically “hot” tumors by modifying the tumor microenvironment (TME) [1].

Currently, most tumor vaccine strategies rely on incorporating tumor rejection antigens (typically peptides or proteins) with adjuvant systems. Newer approaches to tumor vaccination, known as in situ vaccination, rely on strong induction of local tumor immunity, which in turn triggers systemic immunity capable of inducing regression of distant tumors [9,10]. Several different approaches to in situ vaccination have been described, and many involve induction of innate immunity via injection of TLR 3 or 9 ligands, combined with agonistic antibodies to immune stimulatory checkpoint molecules including OX40 and CD40 (reviewed in Saxena et al. [9]). In situ tumor vaccination has also been combined with local RT [11,12].

There is emerging interest in the role of the OX40/OX40L axis as an immune stimulation signal for cancer therapy [13,14,15,16]. OX40 is a co-stimulatory checkpoint molecule belonging to the tumor necrosis factor receptor superfamily that is expressed primarily on CD4+ T cells and regulatory T cells [17]. Through interaction with its ligand (OX40L), OX40 exerts anti-tumor immune effects via facilitating activation and survival of effector T cells and promoting the generation of memory T cells [18,19,20,21,22]. Another proposed mechanism of OX40 immunotherapy is inhibition of the generation and function of regulatory T cells (Tregs) and subsequent removal of the Treg immune suppressive effects within the TME [23,24,25,26,27,28].

It has been demonstrated in in vitro and in vivo preclinical models that radiation induces OX40 ligand expression, leading to expansion of costimulatory signaling through OX40 on T cells [29,30,31,32]. Another approach is to combine anti-OX40 agonistic monoclonal antibody (mAb) with TLR agonists, including TLR3, TLR4, and TLR9 [33]. Low doses of TLR ligands injected intratumorally also induces expression of OX40 on CD4+ T cells, directly activating antigen presenting cells and triggering antigen-specific T cell immune responses [33]. High-dose radiation followed by OX40 stimulation effectively inhibited local and systemic antitumor growth, limited lung metastases, and improved survival rates in a murine model of anti-PD1-resistant lung tumors, while the treatment combination augmented CD4+ and CD8+ T-cell expansion and induced the expression of OX40 on T cells in tumors and spleens [29]. Finally, high-dose radiation in combination with a TLR9 agonist in pre-clinical tumor models enhanced anti-tumor immune responses via increasing activated CD4+ and CD8+ T cells within the tumor microenvironment [34].

Building upon these prior findings, we hypothesized that the immunostimulatory effects of SBRT with in situ tumor vaccination would enhance anti-tumor immune responses compared to SBRT alone. The in situ vaccination approach combined an OX40 agonist antibody with TLR 3 and TLR 9 agonists (polyIC and non-coding plasmid DNA, respectively) complexed to cationic liposomes. The effectiveness of this approach in modifying the TME, which has previously only been evaluated as variations of this combination in rodent models [29,30,31,33,34], was assessed in pet dogs with spontaneous cancers.

Spontaneous cancer in companion dogs is emerging as a valuable model for immunology research as the benefits of studying canine cancer patients for obtaining translational preclinical cancer immunology data are being recognized [35,36]. Companion dogs have an immune system which is well characterized with considerable and meaningful homology to humans. The immune system of companion dogs is immunologically experienced, with most pet dogs having been naturally exposed throughout their lives to viral and bacterial infections, as well as to environmental stressors; dogs also typically receive series of immunizations against pathogens as juveniles and then boosters as adults [37]. Cancer develops in dogs naturally over time; therefore, at the time of diagnosis, their cancer has evolved through the phases of immunosurveillance to immune evasion, over the course of weeks to months of tumor exposure. Further, as dogs are treated for their cancer, their immune system responds naturally with similar shifts between pro-inflammatory anti-tumor responses and tumor promoting immunosuppressive responses within the tumor microenvironment and systemically as has been documented in preclinical rodent-based research and human clinical trials [38,39].

This study performed using a spontaneous canine cancer model is an important translational step to justify future human clinical trials. We randomized dogs with solid tumors (melanoma, carcinoma, soft tissue sarcoma) to treatment with either SBRT alone, or SBRT plus in situ vaccination, and assessed the immunologic effects of these treatments on the TME.

## 2. Results

### 2.1. Combination Radiation Therapy and Immunotherapy Study Design and Animal Enrollment

**Figure 1:**
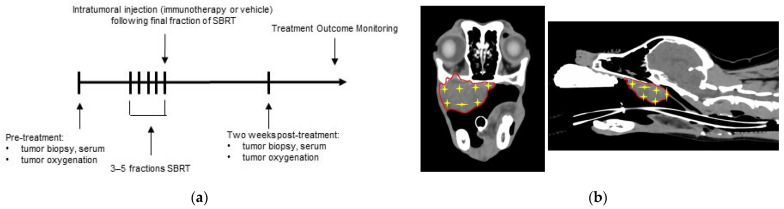
Combination radiation therapy and immunotherapy study design: (**a**) Dogs were randomized into two treatment groups: (1) SBRT only and (2) SBRT and immunotherapy; serial tumor and serum samples were obtained and tumor oxygenation measurements were obtained prior to and two-weeks post treatment. (**b**) Representative images of a three-dimensional injection map which was prepared prior to injections for each dog in order to homogenously inject immunotherapy or vehicle (PBS). Red line outlines gross tumor volume; yellow stars indicate an injection site.

Twelve dogs were enrolled in the study, with six dogs randomized to each treatment group. The study design and injection CT-guided mapping approach are depicted in Figure 1. In the SBRT only treatment group, there were three dogs with melanomas, two with carcinomas, one dog with a soft tissue sarcoma. In the SBRT + Immunotherapy treatment group, there were two dogs with melanomas, two dogs with carcinomas, and two dogs with soft tissue sarcomas. Each animal received SBRT at a dose of 6–10 Gy per fraction for a total of 3–5 fractions each. The RT protocol decisions were based on standard clinical considerations, taking into account tumor type, size, and location The tumor locations, stage of disease, tumor volumes at the onset of treatment, and dose/volume of injections are described in Table 1. There was no difference in gross tumor volume (GTV) between dogs in the SBRT (mean 26.6 cm^3^, range 5.5–66 cm^3^) and SBRT + immunotherapy (mean 47.3 cm^3^, range 2.3–112.7 cm^3^) treatment groups (*p* = 0.298). One dog in the SBRT group was treated with adjuvant chemotherapy (carboplatin), initiated eight days post-RT; one dog in the SBRT + immunotherapy received chemotherapy (carboplatin) ten days prior to SBRT.

### 2.2. Immune Infiltrates and Impact of SBRT and Immunotherapy

As the inherent differences in baseline tumor immune cell populations are highly variable across patients and tumor types, we present the treatment-associated shifts in TME immune cell infiltrates as fold-change, post- relative to pre-treatment, to more accurately reflect the local tumor immune effects. Regulatory T cell (FoxP3+) infiltrates in tumor tissues increased in tumors receiving SBRT only (mean density 0.44% pre-treatment vs. 1.24% post-treatment, fold change: 2.24) whereas Treg density was reduced following treatment in animals treated with SBRT plus immunotherapy (mean density: 0.84% pre-treatment vs. 0.423% post-treatment, fold change: 0.607) (Figure 1). The difference in fold change in Treg density pre- to post-treatment between tumor treated with SBRT and SBRT plus immunotherapy was statistically significant (*p* = 0.008). Tumor CD204+ macrophage infiltrate density increased in dogs receiving SBRT only (mean density: 7.57% pre-treatment vs. 8.79% post-treatment, fold change: 1.51), whereas the density decreased in tumors from animals that received SBRT + immunotherapy (mean density: 24.2% pre-treatment vs. 14.75% post-treatment, fold change: 0.50); the fold change in macrophage density was not significantly different between the treatment groups (*p* = 0.151) (Figure 1).

**Figure 2:**
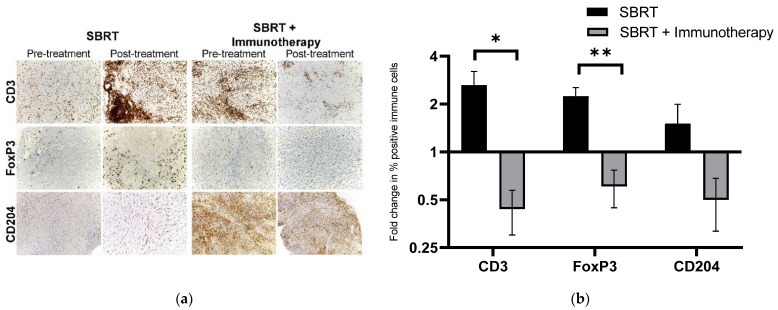
Tumor immune infiltrates associated with SBRT and SBRT + immunotherapy: (**a**) representative IHC images of immune cell infiltrates, pre-treatment and post-treatment, for tumors treated with SBRT or SBRT + immunotherapy; (**b**) fold change in percent positive immune cell density post-treatment relative to pre-treatment for tumors treated with SBRT or SBRT + immunotherapy. * *p* = 0.025, ** *p* = 0.008 (SBRT: n = 4; SBRT and immunotherapy: n = 3).

CD3+ T cell density increased in the SBRT only group (mean density: 3.4% pre-treatment vs. 10.1% post-treatment, fold change: 2.63), whereas paradoxically the CD3+ T cell density decreased in tumors that received SBRT + immunotherapy (mean density 5.3% pre-treatment vs. 3.56% post-treatment, fold change: 0.44) (Figure 1). The difference in fold change in CD3+ T cell density pre- to post-treatment between tumors treated with SBRT and SBRT plus immunotherapy was statistically significant (*p* = 0.025). The Pax5+ B cell infiltrates did not change following SBRT or SBRT plus immunotherapy (fold change, *p* = 0.955) (Figure 2).

### 2.3. Immune Gene Expression in Tumor Tissues and Impact of SBRT and Immunotherapy

**Figure 3:**
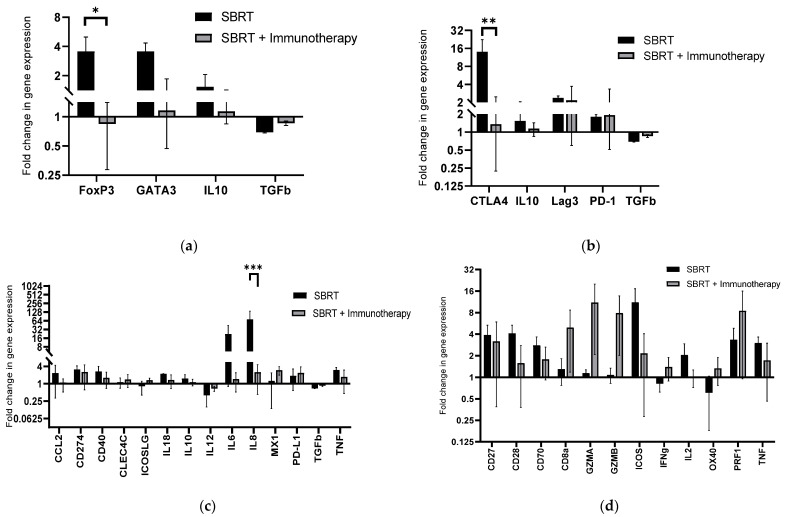
Tumor immune gene expression associated with SBRT and SBRT + immunotherapy: Fold change in post-treatment relative to pre-treatment gene expression counts for tumors treated with SBRT or SBRT and immunotherapy via custom canine Nanostring immune panel. Gene expression profiles were divided into patterns associated with (**a**) regulatory T cell gene expression; (**b**) exhausted T cell gene expression; (**c**) myeloid cell gene expression; (**d**) effector T cell gene expression. * *p* = 0.038, ** *p* = 0.005, *** *p* = 0.0001 (SBRT: n = 2, SBRT + immunotherapy: n = 3).

The impact of SBRT and immunotherapy on immune gene expression was evaluated using a custom Nanostring panel designed for quantification of expression of 45 genes related to cancer immune responses in dogs. Gene expression profiles were divided into patterns associated with Tregs, exhausted T cells, myeloid cells, and effector T cells (Figure 3). A significant difference in the fold-change of the Treg gene FoxP3 was identified in the Treg gene expression profile (Figure 3a) (*p* = 0.0378). Tumors treated with SBRT alone had increased expression of FoxP3 (mean: 52.0 pre-treatment vs. 161.9 post-treatment, fold change: 3.6) and tumors treated with SBRT + immunotherapy had reduced expression of FoxP3 (mean: 246.9 pre-treatment vs. 103.2 post-treatment, fold change: 0.84). The fold-change in expression of CTLA4, a gene associated with negative regulation of T cell activation, was significantly different between the treatment groups in the exhausted T cell gene expression profile (Figure 3b) (*p* = 0.0054). Tumors treated with SBRT alone had significantly greater fold-change in CTLA4 expression (mean: 50.5 pre-treatment vs. 622.3 post-treatment, fold change: 14.0) while this effect was reduced in tumors treated with SBRT + immunotherapy (mean: 338.0 pre-treatment vs. 273.1 post-treatment, fold change: 1.35). There were no significant findings in genes associated with activated myeloid cells (Figure 3c). However, there was a significant decrease in the expression of IL-8 in tumors post-SBRT + immunotherapy compared to SBRT alone (*p* = 0.0001) (Figure 3c). IL-8 is associated with immunosuppressive myeloid cells. Tumors treated with SBRT alone had increased expression of IL-8 (mean: 16,950.0 pre-treatment vs. 122,804.7 post-treatment, fold change: 72.6), while this effect was reduced in tumors treated with SBRT + immunotherapy (mean: 723.8 pre-treatment vs. 1445.1 post-treatment, fold change: 2.5). No significant differences in effector T cell gene expression were revealed between treatment groups. We did, however, find that effector cell genes CD8a, GZMA, GZMB, IFNγ, OX40, and PRF-1 all had relatively increased fold change in expression following SBRT + immunotherapy compared to SBRT alone (Figure 3d).

### 2.4. Serum Cytokine Responses Following Treatment with SBRT or SBRT and Immunotherapy

**Figure 4:**
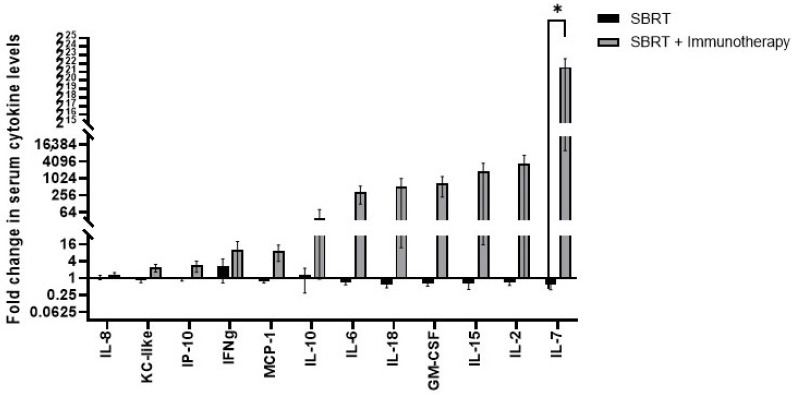
Serum cytokine responses associated with SBRT or SBRT and immunotherapy. Fold change in serum cytokine levels post-treatment relative to pre-treatment for dogs treated with SBRT or SBRT + immunotherapy. * *p* = 0.035 (SBRT n = 6, SBRT and immunotherapy n = 6).

Of the 13 cytokines assessed, only the fold change in serum IL-7 concentrations from pre- to post-treatment were significantly different between dogs treated with SBRT alone compared to SBRT plus immunotherapy (Figure 4). The fold change in serum IL-7 concentrations was significantly higher in animals treated with SBRT plus immunotherapy compared to SBRT only animals (SBRT fold change: 0.588 vs. SBRT + immunotherapy fold change: 3.14 × 10^6^, *p* = 0.035) (Figure 4).

### 2.5. Impact of SBRT or SBRT and Immunotherapy on Tissue Vascularity and Oxygenation

**Figure 5:**
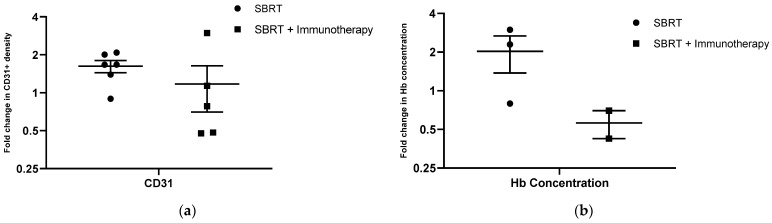
Tissue vascularity and oxygenation associated with SBRT or SBRT and immunotherapy. (**a**) Fold change in density of CD31+ endothelial cells in post-treatment relative to pre-treatment samples for dogs treated with SBRT or SBRT + immunotherapy (SBRT n = 6, SBRT and immunotherapy n = 5); (**b**) fold change in tumor hemoglobin saturation post-treatment relative to pre-treatment. (SBRT n = 3, SBRT and immunotherapy n = 2).

As previous studies have illustrated the impact of radiation therapy on tumor vascularity and how these responses may be modified by immunotherapy [40,41], we evaluated tissue vascularity and oxygenation parameters pre- and post-treatment in a subset of dogs in this study. To evaluate changes in the vascularity of the irradiated tissue microenvironment, we analyzed tumor biopsy samples pre- and post-treatment regardless of the percentage of viable tumor tissue in the section. However, one melanoma case in the SBRT and immunotherapy group was excluded from analysis due to substantial melanin pigmentation confounding the IHC results. Tissue biopsies from sites treated with SBRT alone showed a mean 1.62-fold increase in CD31+ cell density. The fold increase in CD31+ cells in tissue biopsies treated with SBRT and immunotherapy was 1.17. However, this difference in fold change in CD31+ density was not statistically significant between treatment arms (*p* = 0.515) (Figure 5a). There was no change in hemoglobin saturation in tumors treated with either SBRT (mean fold change: 1.01) or SBRT plus immunotherapy (mean fold change: 1.02) from pre-treatment to the two-week time point (*p* = 0.344) (Figure 5b). However, we noticed a trend with respect to tumor hemoglobin concentration, as tumors treated with SBRT had a relative increase in Hb concentration at the two-week time point (mean fold change: 2.03) while tumors treated with SBRT and immunotherapy had a relative decrease in hemoglobin concentration at the two-week time point (mean fold change: 0.56) (Figure 5b). The difference in changes in hemoglobin concentration between the treatment groups did not reach statistical significance (*p* = 0.15) (Figure 5b).

### 2.6. Treatment Responses to SBRT and SBRT and Immunotherapy

The treatment response and outcome results of dogs evaluated in the study are summarized in Table 2. It should be noted that the study was not designed to assess the therapeutic efficacy of adding OX40/TLR agonist immunotherapy to SBRT, since only small numbers of animals were enrolled and only a single immunotherapy treatment was administered. Briefly, dogs treated with SBRT alone experienced a median PFS of 137 days (range: 75–260 days) and dogs treated with SBRT + immunotherapy had a median PFS of 98 days (range: 19–433 days); dogs treated with SBRT alone had a median OST of 357 days (range: 147–477 days) and dogs treated with SBRT + immunotherapy had a median OST of 208 days (range: 52–635 days). No statistically significant differences were found between treatment responses (*p* = 0.567), PFST (*p* = 0.575), and OST (*p* = 0.943) for dogs treated with SBRT compared to SBRT + immunotherapy.

Acute toxicity was identified in one dog in the SBRT alone group (n = 1/6, 16.7%), consisting of Grade 3 mucosal ulceration and bone exposure. Acute toxicity was identified in one dog in the SBRT + immunotherapy group (n = 1/6, 16.7%), consisting of Grade 1 esophagitis and Grade 3 laryngitis/tracheitis; the acute toxicities for this dog resolved with medical management. Delayed toxicity was identified in the SBRT alone group and consisted of Grade 3 osteoradionecrosis in two dogs (n = 2/6, 33%); one of these dogs had acute bone exposure that persisted into the delayed toxicity time frame, and the other dog developed osteoradionecrosis 99 days following SBRT. Two dogs in the SBRT + immunotherapy group developed delayed Grade 1 skin toxicity (n = 2/6, 33%) that persisted as late toxicity consisting of alopecia and leukotrichia of the skin in the radiation field. There were no statistical differences in the toxicity profiles across dogs in either treatment group (acute: *p* > 0.99, delayed/late: *p* > 0.99, incidence of severe toxicity: *p* > 0.99, incidence of any toxicity: *p* > 0.99).

## 3. Discussion

In this project, we evaluated the immunologic effects of SBRT in spontaneous canine tumors, as well as the impact of in situ vaccination with OX40/TLR3/9 immunotherapy. The combination of SBRT and local OX40/TLR3/9 immunotherapy was well-tolerated by study animals, with a similar toxicity profile as those treated with SBRT alone. Importantly, the addition of OX40/TLR3/9 immunotherapy to SBRT resulted in local depletion of Tregs and reduced Treg-associated gene expression (FoxP3), reduced the density of tumor-associated macrophages and suppressed macrophage-associated gene expression (IL-8), suppressed exhausted T cell-associated gene expression (CTLA4), and induced an increase in circulating IL-7 concentrations compared to dogs treated with SBRT alone. However, there was also a paradoxical decrease in the density of effector CD3+ T cells in tumor tissues that received combined SBRT and immunotherapy as compared to animals treated with SBRT only.

This study allowed for evaluation of the immunologic effects of SBRT on the TME in spontaneous canine tumors. This was an important aspect of the study, since, to our knowledge, there has been limited published evidence of the effects of SBRT on local immune responses in the TME in humans [42] and no prior publications of these effects in spontaneous large animal cancer models. The biological effects of RT vary according to radiation dosing and dose per fraction [35]. For example, in mouse cancer models, hypofractionated or single high dose RT treatments comparable to SBRT protocols have resulted in increases in tumor infiltrating lymphocytes [43]. Conversely, these high-dose RT protocols also increase immunosuppressive properties of the TME, such as expansion of Tregs [44], tumor associated macrophages [45], and MDSCs [45,46]. For human cancer patients, post-SBRT-associated TME immunological changes have been reported in a pilot study involving SBRT for patients with renal cell carcinoma. This study observed that Ki67+-proliferating CD8+ T cells and FOXP3+ Tregs were increased in tumor samples four weeks following irradiation [42]. Interestingly, in our study we also observed that SBRT alone resulted in increased tumor densities of CD3+ T cells and FoxP3+ Tregs, as well as CD204+ macrophages. Additionally, canine tumors treated with SBRT had increased expression of genes associated with an immunosuppressive TME, notably the Treg-associated gene FoxP3, CTLA4, a gene associated with negative regulation of T cell activation, and IL-8, associated with recruitment of immunosuppressive myeloid cells. These results generated from a spontaneous large animal model illustrate the complexities associated with understanding the immunological impacts of SBRT on the TME. As radiotherapy can both enhance immune activation and cause immune suppression, efforts are underway to decipher and optimize RT parameters necessary to achieve effective immunogenic modulation of the TME [35].

In situ tumor vaccination with intratumoral injection of OX40 antibodies in combination with TLR3 or TLR9 agonists has been reported in rodent models to induce systemic antitumor immunity [33]. Importantly, variations of this treatment approach are now under investigation clinically in human trials (NCT03410901, NCT04387071). Our proof-of-concept study revealed an important impact of the combined treatment on the TME, namely significant Treg depletion. Treg depletion has been reported previously as a primary mechanism driving the anti-tumor activity of systemic OX40 antibody immunotherapy [27], but this is the first report of a local effect of Treg depletion and reduced Treg-associated gene expression with the combination of SBRT, OX40 agonistic antibody, and TLR3/9 agonists in naturally occurring tumors.

There was a significant difference in the fold change of circulating concentrations of IL-7 documented in the dogs of this study treated with SBRT and immunotherapy compared to dogs treated with SBRT alone. Increased circulating levels of IL-7 promote lymphocyte development in the thymus and maintain homeostasis of peripheral naïve and memory CD4 T cells [47]. At this time, the biological and clinical impact of this finding is unknown but will be investigated further in future studies. Notable increases in the fold change of circulating levels of IL-2, IL-15, and IL-18 were also documented following SBRT and immunotherapy; these cytokines have been associated with promising cancer immunotherapeutic approaches [48,49,50].

The addition of OX40/TLR3/9 immunotherapy to SBRT also resulted unexpectedly in T cell depletion from tumor tissues relative to animals treated with SBRT alone. At present it is difficult to fully explain this response, and it may have therapeutic implications. However, it is also possible that the apparent T cell depletion effect is transient and may rebound at later time points after SBRT + immunotherapy. Additional studies with longer tumor sampling periods may help fully elucidate the response.

Finally, we evaluated treatment effects on tumor vascularity and oxygenation parameters. We observed a trend whereby tumors treated with SBRT had a greater increase in both endothelial cell density and hemoglobin concentration at the two-week timepoint compared to tumors treated with SBRT and immunotherapy. However, evaluating these factors in a larger study with more animals would be necessary to fully understand these effects. It has been demonstrated in preclinical models [51,52] and clinically [53] in head and neck cancer patients that radiation-resistant tumors reoxygenate in response to radiation. As such, we plan to continue to further investigate treatment effects on tumor vascularity and oxygenation in future studies.

In summary, these results obtained in a spontaneous large animal cancer model indicate that addition of OX40/TLR immunotherapy to SBRT exerts important immunological effects both locally (Treg, macrophage, and T cell depletion) and systemically (increased circulating IL-7 concentrations). While not designed to assess therapeutic efficacy, these early findings provide the impetus and rationale for additional follow-on studies. In particular, a therapeutic trial would be designed to include multiple local tumor in situ vaccination treatments to follow the SBRT protocol, to sustain and expand the systemic immunological effects. In addition, studies with larger numbers of dogs, with a more homogenous tumor population would help reduce animal-to-animal variability, and a standardized SBRT protocol would be used. Such a study would also include measures of induction of systemic as well as local anti-tumor immunity, with correlative studies of immune responses and treatment outcomes. Additionally, a deeper evaluation of treatment-associated changes in gene expression within the TME would be performed, involving pathway enrichment analysis, to stimulate exploration into the underlying mechanisms of local and systemic effects. The canine spontaneous cancer model is thus well-positioned to address clinically relevant questions that can aid in the design and implementation of new human trials of combination RT/immunotherapy.

## 4. Materials and Methods

### 4.1. Study Design

All experimental protocols were reviewed and approved by the Colorado State University (CSU) Institutional Animal Care and Use Committee and the Clinical Review Board (IACUC #639, VCS #2019-194). Informed consent was obtained from all clients prior to enrollment of their dogs into the trial. Dogs presented to the Flint Animal Cancer Center of the Colorado State University Veterinary Teaching Hospital were considered for enrollment if they had a solid external tumor amenable to SBRT, local tumor injection, and serial tumor biopsies. Dogs with tumors arising from bone or affecting regional bone(s) with moderate to high risks of fracture with treatment and/or biopsies were not eligible for enrollment, as well as dogs with tumors with high risks for hemorrhage with sampling. For enrollment, tumors had to be <150 cm^3^. Chemotherapy was prohibited within 7 days of SBRT +/− immunotherapy. Dogs were randomized and into two treatment groups: 1) SBRT + local injection of vehicle control or 2) SBRT + local injection of immunotherapy, with efforts to distribute tumor types evenly between the treatment groups. Pre-treatment tumor tissue and serum samples were collected (Figure 1). Dogs were anesthetized and treated with SBRT, as prescribed by the attending veterinary radiation oncologist (Varian Eclipse treatment planning system^TM^, Varian, Palo Alto, CA, USA) and delivered via 6 MV photon radiation (Varian Trilogy linear accelerator, Varian, Palo Alto, CA, USA). Intratumoral injections were administered following the final fraction of SBRT. Dogs treated with immunotherapy received independent injections of a canine OX40-specific agonistic monoclonal antibody (Chow, L., et al.; manuscript in preparation) and cationic liposomes complexed to equal amounts by weight of a TLR3 agonist (polyIC, InVivoGen, San Diego, CA, USA) and non-coding plasmid DNA (pDNA) as TLR9 agonist [54,55,56]. The dose and volume of injections administered were based on tumor size (OX40 antibody: tumor < 5 cm^3^ = 100μg in 1 mL, tumor 5–10 cm^3^ = 150 μg in 2 mL, tumor > 10 cm^3^ = 200 μg in 3 mL). The TLR3/9 ligand injection was also scaled according to tumor size, with the following 3 doses administered: tumor < 5 cm^3^ = 5 μg polyIC/5 μg pDNA in 1 mL, tumor 5–10 cm^3^ = 7.5 μg polyIC/7.5 μg pDNA in 2 mL, tumor > 10 cm^3^ = 10 μg polyIC/10 μg pDNA in 3 mL. Dogs in the SBRT only group had tumors injected with PBS, using the same tumor size scale to determine injection volumes. Tumor injection maps were prepared prior to injection, using the simulation CT scan for SBRT treatment planning in order to obtain uniform injection throughout the tumor (Figure 2). Post-treatment tumor tissue biopsies and serum samples were collected two weeks after completion of SBRT (Figure 1).

### 4.2. Tissue Sampling

Two tumor samples were obtained from each dog at pre- and two weeks post-SBRT time points with either a 4–10 mm punch biopsy or wedge biopsy; samples were acquired and distanced from a previously biopsied site to avoid collecting tissues with prior local TME disruption. One tumor sample was placed in 10% neutral buffered formalin and the other in RNA*later* stabilization solution (Invitrogen, Waltham, MA, USA) and flash-frozen in liquid nitrogen. Frozen tissues were stored at −80 °C and formalin-fixed samples at 4 °C until processed for histology. To collect serum at each time point, 2–3 mL of whole blood was obtained and allowed to clot for 5–15 min. The blood samples were then centrifuged, and the serum was collected and stored at −20 °C until processed.

### 4.3. Histological and Immunohistochemical Evaluation and Quantitative Image Analysis

Formalin fixed tissue samples were paraffin embedded, sectioned, and stained with hematoxylin and eosin. Hematoxylin and eosin slides were reviewed by a board-certified pathologist (DPR) to confirm diagnosis and assess for the presence of viable tumor tissue for immunohistochemical labeling. Slides with minimal to no tumor tissue present were excluded from analysis. Immunohistochemistry was performed using a Leica Bond Max autostainer (Leica Biosystems Inc., Wetzlar, Germany), with the following panel of previously published canine cross-reactive primary antibodies directed against the following antigens/cell types, at the listed concentrations: monoclonal mouse anti-human CD3 (pan T lymphocyte marker; Leica Biosystems Inc., Wetzlar, Germany, clone LN10; 10 μg/mL), monoclonal mouse anti-human CD204 (macrophages; TransGenic Inc. Tenjin, Fukuoka, Japan, clone SRA-E5; 1.25 μg/mL), mouse monoclonal anti-human Pax5 (B lymphocytes; Leica clone IEW; ready-to-use format), mouse monoclonal anti-human FoxP3 (regulatory T cells; ThermoFisher, Waltham, MA, USA, clone eBio7979; 5 μg/mL), and mouse monoclonal anti-human CD31 (endothelial cells; Leica, clone JC70A; ready-to-use format). Deparaffinization and rehydration was performed on the Bond autostainer using a series of xylenes and graded ethanols. Antigen retrieval was performed using either: Leica Epitope Retrieval 2 (Tris-EDTA buffer, pH 9; CD3, CD204, FoxP3, CD31), or Leica Epitope Retrieval 1 (Citrate buffer, pH 6; CD79a), both for 20 min at 100 °C. Detection was performed with PowerVision IHC detection systems (Leica Biosystems, Inc, Leica Biosystems Inc., Wetzlar, Germany), using a polymeric horseradish peroxidase anti-mouse IgG and Bond Polymer Refine DAB or Red chromogen, with routine hematoxylin counterstain.

For quantitative analysis of immune cell density, whole slide brightfield images of IHC stained slides were digitally captured using an Olympus VS120 slide scanner at 20× magnification and fixed exposure times for all samples. Quantitative image analysis was performed using Visiopharm software (Visiopharm Corporation, Hovedstaden, Denmark). Briefly, a positive pixel threshold for all immune cell markers was determined using positive control and corresponding isotype-stained control slide images and visually confirmed by a veterinary pathologist. Images were subjected to universal application of this intensity threshold to all images. Following image analysis, pseudo-colored positive pixel masks of each image were evaluated by a pathologist to ensure proper thresholding and accuracy of the analysis algorithm for immune cell marker detection. Data were analyzed and the number of infiltrating immune cells was expressed as immune cell positive area as a percentage of total tissue area analyzed. The total tissue region of interest for each analyzed slide was limited to viable tumor and adjacent tumor-associated stroma and was manually annotated to include this tissue while excluding artifacts such as hemorrhage, necrosis, tissue-folds, or other sectioning artifacts. Data were analyzed and the number of infiltrating immune cells was expressed as a percentage of this total tumor-associated tissue area. Tumors with adequate pre- and post-treatment tumor samples were included in analyses of fold change in immune cell density.

### 4.4. Gene Expression Analysis

Frozen tumor samples were processed for gene expression analysis if the corresponding FFPE samples for that time point was determined to have >50% tumor tissue present on histopathologic review. RNA was extracted from frozen tumor tissues using the RNeasy Plus Mini kit (QIAGEN, Hilden, Germany) following manufacturer protocol. Depending on starting material, samples were eluted in 30–50 μL RNase-Free water. Samples were initially checked for quantity and purity on a Nanodrop ND-1000 Spectrophotometer (Thermo Fisher, Waltham, MA, USA) prior to being stored at −80 °C until further processing. Samples were additionally quantity and quality checked using the RNA High Sensitivity assays on the Qubit 2.0 Fluorometer (Invitrogen/LifeTechnologies, Carlsbad, CA, USA) and 5200 Fragment Analyzer Automated CE System (Agilent, Santa Clara, CA, USA), respectively. NanoString (NanoString Technologies, Seattle, WA, USA) gene expression analysis was performed using a custom-designed 48 gene canine immune panel derived from Rooney et al. [57] (Supplementary Table S1). The genes included in this panel are listed in Supplementary Table S1. Nanostring analysis was performed with the nCounter Analysis FLEX system at the University of Arizona Genetics Core. Gene expression count data were analyzed via nSolver software (NanoString Technologies, Seattle, WA, USA). Tumors with adequate pre- and post-treatment tumor samples were included in analyses of fold change in gene expression.

### 4.5. Serum Cytokine Analysis

Serum samples were evaluated for cytokine levels using MILLIPLEX Canine Cytokine/Chemokine Magnetic Bead Panel (MilliporeSigma, Burlington, MA, USA, CCYTOMAG-90K) according to the manufacturer’s instructions. Samples were run on a Luminex MAGPIX multiplex instrument and data were analyzed using Luminex xPONENT software (Luminex Corporation, Austin, TX, USA). If the pre-treatment cytokine level for a given cytokine was undetectable, the cytokine was excluded the from group fold change analyses.

### 4.6. Tumor Oxygenation

A non-invasive optical spectroscopy system (Zenascope, Zenalux Biomedical, Durham, NC, USA) was used to measure tumor tissue hemoglobin saturation and hemoglobin concentration. Three to five measurements were obtained from distanced sites across the tumor volume pre-treatment and at the two-week post-treatment timepoint.

### 4.7. Determination of Tumor Responses, Progression Free Survival and Overall Survival Times

Follow-up time was calculated from the first day of SBRT to the last day of follow-up. Evaluation of tumor response was based on Response Evaluation Criteria in Solid Tumor (RECIST) in dogs as complete response (CR), partial response (PR), stable disease (SD), or progressive disease (PD) [58]. To be characterized as achieving CR, PR, or SD, the treatment response must have persisted for at least three months. Progression free survival (PFS) and overall survival time (OST) were calculated from the first day of SBRT to the day of disease progression or death. Patients were censored if lost to follow-up or still alive at the end of the study.

### 4.8. Toxicity Grading Criteria

Normal tissue toxicity was graded according to Veterinary Radiation Therapy Oncology Group (VRTOG) and Radiation Therapy Oncology Group (RTOG) radiation morbidity scoring scheme according to review of medical records (CSU and primary veterinary practices) [59,60]. Radiation adverse events were defined as acute (within 90 days after radiation therapy), delayed (90 days to 6 months after radiation therapy), or late toxicity (greater than 6 months after radiation therapy).

### 4.9. Statistical Analysis

All statistical analyses were performed using commercial software (Prism 8; GraphPad Software, Inc., San Diego, CA, USA). Continuous data are expressed as mean ± standard error of the mean (SEM). Differences in means between treatment groups were compared by two-tailed unpaired parametric t-test. Two-way analysis of variance (ANOVA) followed by Sidak’s multiple comparisons test was used to evaluate differences across multiple independent factors between treatment groups for the fold change in gene expression data and cytokine levels. Kaplan-Meier survival curves of PFS and OST were created, and median PFS (MPFS) and MST were compared by log rank test. RECIST treatment response was examined by Fisher’s Exact test. For all analyses, the statistical significance level was defined as alpha < 0.05.

## Figures and Tables

**Table 1:** Signalment, disease characteristics, and treatment conditions of study dogs.

Signalment	Tumor Type and Location	Tumor Volume	Injection Type and Volume	Stage of Disease at Time of Treatment	SBRT Protocol
12y MC Australian Terrier	Melanoma (mandible)	5.5 cm^3^	Vehicle control (PBS) (2 mL injections)	Primary tumor	10 Gy × 3
13y FS Mixed Breed	Carcinoma (salivary gland)	66 cm^3^	Vehicle control (PBS) (3 mL injections)	Primary tumor, Distant (pulmonary) metastasis	10 Gy × 3
10y MC Labrador Retriever	Melanoma (maxilla)	51.9 cm^3^	Vehicle control (PBS) (3 mL injections)	Primary tumor	10 Gy × 3
3y MC German Shepherd	Soft tissue sarcoma (mandible)	8.6 cm^3^	Vehicle control (PBS) (2 mL injections)	Primary tumor	6 Gy × 5
9y FS Maltese	Melanoma (maxilla)	17.9 cm^3^	Vehicle control (PBS) (3 mL injections)	Primary tumor	10 Gy × 3
15y MC Mixed Breed	Carcinoma (maxilla)	9.8 cm^3^	Vehicle control (PBS) (2 mL injections)	Primary tumor	10 Gy × 3
13y FS Miniature Dachshund	Carcinoma (salivary gland)	30.1 cm^3^	OX40/TLR agonists (200 mg OX40 in 3 mL; 10 mg polyIC/10 mg pDNA in 3 mL)	Primary tumor, Regional (nodal) metastasis	8 Gy × 5
8y MC Mixed Breed	Soft tissue sarcoma (maxilla)	112.7 cm^3^	OX40/TLR agonists (200 mg OX40 in 3 mL; 10 mg polyIC/10mg pDNA in 3 mL)	Primary tumor	8 Gy × 5
10y MC Labrador Retriever	Melanoma (maxilla)	2.3 cm^3^	OX40/TLR agonists (100 mg OX40 in 1 mL; 5 mg polyIC/5 mg pDNA in 1 mL)	Primary tumor	10 Gy × 3
10y MC Mixed Breed	Melanoma (maxilla)	33.8 cm^3^	OX40/TLR agonists (200 mg OX40 in 3 mL; 10 mg polyIC/10 mg pDNA in 3 mL)	Primary tumor	10 Gy × 3
10y FS Labrador Retriever	Carcinoma (mandible)	35.5 cm^3^	OX40/TLR agonists (200 mg OX40 in 3 mL; 10 mg polyIC/10 mg pDNA in 3 mL)	Primary tumor	10 Gy × 3
9y FS Miniature Poodle	Soft tissue sarcoma (axilla)	69.9 cm^3^	OX40/TLR agonists (200 mg OX40 in 3 mL; 10 mg polyIC/10 mg pDNA in 3 mL)	Primary tumor	8 Gy × 5

MC: male castrated, FS: female spayed.

**Table 2:** Treatment response and survival data for study dogs.

Signalment ^1^	Tumor Type and Location	Treatment Response	Progression Free Survival (Days)	Pattern of Failure	Overall Survival Time (Days)	Cause of Death
12y MC Australian Terrier	Melanoma (mandible)	Progressive disease	75	Local, Regional, Distant	452	Local, Regional, Distant
13y FS Mixed Breed	Carcinoma (salivary gland)	Stable disease	115	Distant	477	Local, Regional, Distant
10y MC Labrador Retriever	Melanoma (maxilla)	Complete response	147	Local	147	Local
3y MC German Shepherd	Soft tissue sarcoma (mandible)	Partial response	127	Local	262	Local
9y FS Maltese	Melanoma (maxilla)	Complete response	260	Local	371 (alive at the time of analysis)	Alive at the time of analysis
15y MC Mixed Breed	Carcinoma (maxilla)	Complete response	154	Local	162	Local
13y FS Miniature Dachshund	Carcinoma (salivary gland)	Progressive disease	19	Regional (out-of-field)	52	Regional (out-of-field)
8y MC Mixed Breed	Soft tissue sarcoma (maxilla)	Stable disease	114	Distant	224	Distant
10y MC Labrador Retriever	Melanoma (maxilla)	Partial response	260	Local, regional (nodal), distant	635	Local, Regional, Distant
10y MC Mixed Breed	Melanoma (maxilla)	Partial response	82	No evidence of progression at time of death	82	Other (Acute collapse)
10y FS Labrador Retriever	Carcinoma (mandible)	Progressive disease	79	Regional (out-of-field)	192	Other (Acute kidney injury)
9y FS Miniature Poodle	Soft tissue sarcoma (axilla)	Stable disease	433 (no progression)	No evidence of progression at time of analysis	433	Alive at the time of analysis

^1^ MC, male castrated; FS, female spayed.

## Data Availability

Complete Nanostring gene expression are data available upon request.

## Supplementary Materials

The following are available online at https://www.mdpi.com/article/10.3390/ijms23020826/s1.
