# Supplementary material for: Immunologic Effects of Stereotactic Body Radiotherapy in Dogs with Spontaneous Tumors and the Impact of Intratumoral OX40/TLR Agonist Immunotherapy
Source: Int J Mol Sci. 2022 Jan 13;23(2):826. doi: 10.3390/ijms23020826 (PMC8775899; doi:10.3390/ijms23020826)
Supplement: Supplementary file 1 [file ijms-23-00826-s001.zip › ijms-1530854-supplementary.pdf]

International Journal of
*Molecular Sciences*

MDPI

*Article*

# Immunologic Effects of Stereotactic Body Radiotherapy in Dogs with Spontaneous Tumors and the Impact of Intratumoral OX40/TLR Agonist Immunotherapy

**Mary-Keara Boss [1,*], Remy Watts [2], Lauren G. Harrison [1], Sophie Hopkins [3], Lyndah Chow [3], Erin Trageser [1], Carina Easton [4], Susan M. LaRue [1], Daniel Regan [4], Mark W. Dewhirst, [5] and Steven Dow [3]**

1 Department of Environmental Health and Radiological Sciences, Colorado State University, Fort Collins, CO 80523, USA; lauren.harrison@colostate.edu (L.G.H.); erin.trageser@colostate.edu (E.T.); susan.larue@colostate.edu (S.M.L.)
2 Department of Companion Animals, Atlantic Veterinary College, Charlottetown, PE C1A 4P3, Canada; rwatts5391@upei.ca
3 Department of Clinical Sciences, Colorado State University, Fort Collins, CO 80523, USA; leone.hopkins@colostate.edu (S.H.); Lyndah.chow@colostate.edu (L.C.); steven.dow@colostate.edu (S.D.)
4 Department of Microbiology, Immunology, and Pathology, Colorado State University, Fort Collins, CO 80523, USA; carina.easton@colostate.edu (C.E.); daniel.regan@colostate.edu (D.R.)
5 Department of Radiation Oncology, Duke University Medical Center, Durham, NC 27710, USA; mark.dewhirst@duke.edu
* Correspondence: keara.boss@colostate.edu

| Gene Name |
|---|
| BCL6 |
| CCL2 |
| CD27 |
| CD274 |
| CD28 |
| CD3e |
| CD40 |
| CD70 |
| CD8a |
| CLEC4C |
| CTLA4 |
| CXCL2 |
| FOXP3 |
| GATA3 |
| GPR146 |
| GZMA |
| GZMB |
| HLA-A |
| ICOS |
| ICOSLG |
| IFNg |
| IL 18 |
| IL10 |
| IL12 |
| IL2 |
| IL6 |
| IL8 |
| KDM6B |
| LAG3 |
| MX1 |

| |
|---|
| NCR1 |
| OX40 |
| Pax5 |
| PD-1 |
| PDCD1LG2 |
| PD-L1 |
| PRF1 |
| RORC |
| SELP |
| TBX |
| TGFb |
| TNF |
| TNFSF10 |
| TNFSF14 |
| ALAS1 (Housekeeping) |
| GUSB (Housekeeping) |
| RPL30 (Housekeeping) |
| SDHA (Housekeeping) |
